# Inhaled Medication Errors During Hospitalization or on Hospital Discharge in Patients Living With Chronic Obstructive Pulmonary Disease: A Literature Review

**DOI:** 10.7759/cureus.48631

**Published:** 2023-11-10

**Authors:** Paul M Boylan, Macie J Gibbs, Katelyn N Helwig

**Affiliations:** 1 Department of Pharmacy: Clinical and Administrative Sciences, The University of Oklahoma Health Sciences Center College of Pharmacy, Oklahoma City, USA; 2 College of Pharmacy, The University of Oklahoma Health Sciences Center, Oklahoma City, USA

**Keywords:** transitional care, patient discharge, hospitalization, medication error, vaporizer, nebulizer, chronic obstructive pulmonary disease

## Abstract

Inhaled medications, including beta-agonists, muscarinic antagonists, and corticosteroids, are the backbone of chronic obstructive pulmonary disease (COPD) treatment, and pharmacotherapy plans are frequently optimized during and following hospitalization. Clinical practice guidelines acknowledge that patients living with COPD may experience medication errors from inadequate inhaler technique or device faults, but inhaled medication errors within COPD pharmacotherapy plans remain unreported. This literature review aimed to collect and present studies describing medication errors occurring with inhaled medications in patients living with COPD during and following hospitalization. The databases searched included Ovid MEDLINE, Embase, and International Pharmaceutical Abstracts. One hundred forty-five unique studies were collected, and 10 studies were included. The rate of inhaled medication errors reported across the 10 studies ranged between 2.5% and 66% of patients living with COPD and who were hospitalized or discharged. The incidence and types of medication errors reported across the studies varied significantly. Standardization in categorizing and reporting inhaled medication errors is necessary for future studies to determine the true incidence of inhaled medication errors occurring in patients living with COPD who are hospitalized or discharged.

## Introduction and background

Patients living with chronic obstructive pulmonary disease (COPD) may take medications to palliate symptoms, improve health status, tolerate exercise, and reduce the risk and severity of exacerbations of COPD (ECOPD) [1]. Inhaled bronchodilators, including short- and long-acting β agonists (SABAs and LABAs, respectively) and short- and long-acting muscarinic antagonists (SAMAs and LAMAs, respectively), represent the backbone of therapy to treat chronic COPD [1]. An inhaled corticosteroid (ICS) may also be added to the pharmacotherapy plan if the patient living with COPD possesses eosinophilia or comorbid asthma [1]. The 2023 Global Initiative for Obstructive Lung Disease (GOLD) Report lists 38 medications across five distinct pharmacologic classes to treat COPD [1]. Fifteen of 38 medications include combinations of inhaled medications (ie, SABA/SAMA, LABA/LAMA, LABA/ICS, and LABA/LAMA/ICS). Single-agent and combination products may be formulated as dry powder, soft mist, or metered dose inhalers or administered via nebulizer [1]. The availability of several devices and the combination of products are advantageous for patients and enable them to use a product that is convenient, affordable, and efficacious.

However, the comprehensive formulary of inhaled medications may be overwhelming for both patients and prescribers and thus there is potential for medication errors. Hospitalization is a critical time wherein inhaled medication errors may occur and between 7% and 33% of patients living with COPD will experience at least one COPD-related hospitalization annually [2]. Patients hospitalized with ECOPD often require pharmacotherapy intensification, such as adding another inhaled bronchodilator (ie, LABA plus LAMA) or inhaled corticosteroid (ie, ICS plus LABA/LAMA), to better manage symptoms and reduce the risk for subsequent ECOPD [1]. For example, duplicate therapies may be erroneously prescribed (i.e., SABA via metered dose inhaler plus SABA via nebulizer). The 2023 GOLD Report acknowledges that medication errors occur, albeit as errors owing to poor inhaler technique or device delivery faults, not necessarily medication errors within the inhaled pharmacotherapy plan itself [1]. Medication errors are defined as mistakes occurring throughout the stages of the medication-use process including medication selection, procurement, storage, ordering, transcribing, preparing, dispensing, administering, and monitoring [3]. Thus, medication errors may encompass either patient or healthcare errors depending on the phase of the medication-use process [3]. Medication errors that occur due to inappropriate prescribing or during the transition of care from hospital to non-hospital phases of care are unacknowledged within the 2023 GOLD Report.

The purpose of this review is to collect and present literature describing medication errors occurring with inhaled medications in patients living with COPD during and following hospitalization.

## Review

Review methods

This was a literature review utilizing the Preferred Reporting Items for Systematic Reviews and Meta-Analysis (PRISMA) protocol [4]. The research question did not satisfy the technical requirements for a systematic review (to answer a specific patient-intervention-comparison-outcome (PICO) question) or a scoping review (to inform policymakers or posit gaps in practice, research, or policymaking), thus a literature review adhering to PRISMA criteria was employed [4]. The research protocol was not registered because literature reviews are ineligible for registration in the International Prospective Register of Systematic Reviews (PROSPERO).

An electronic literature search was performed using Ovid MEDLINE, Embase, and International Pharmaceutical Abstracts on February 14, 2023. Search strings for each database are included in Appendix A and adhered to the PRISMA-S extension for literature search reporting [5]. Study registries were not searched, print resources were not browsed, and in-article citation searching was not performed to collect records beyond the database searches. Articles published between 1946 and 2022 were eligible for inclusion. Exact keywords and filters varied depending on the database searched but consistent terms across the searches were COPD, medication error, and discharge. Studies were eligible for inclusion if they were published in English and the population studied included patients living with COPD, were taking or prescribed inhaled medication(s) to treat COPD, and were hospitalized or recently discharged from the hospital. We did not aim to capture, characterize, and assess errors in inhaler administration because inhaler technique errors are already and clearly described in the GOLD Report [1].

Results from each database search were exported as a Research Information Systems file and then imported into Covidence (Covidence, Melbourne, AU). During the initial file upload, Covidence automatically de-duplicated identical records retrieved across databases. Abstracts were independently screened by two authors (KH and MG) and screening conflicts were resolved by a third author (PB). Full texts were independently screened by two authors (KH and MG) and screening conflicts were resolved by a third author (PB). Data abstraction was independently performed by two authors (KH and MG). To minimize heterogeneity across the articles and among authors, extraction was performed using a template in Covidence. Data collected included study location, study design, characteristics of the cohort (of all subjects included in the research), characteristics of patients living with COPD (i.e., a sub-group if the research reported on multiple acute or chronic diseases beyond COPD), and medication errors. There are many frameworks for reporting medication errors, medication error research, and outcomes attributable to medication errors [3]. Because of this literature review design and the expected heterogeneity across included studies, we extracted medication errors as reported in each of the included studies using counts and frequencies, and utilized narrative synthesis to briefly summarize prescient outcomes in this manuscript [4]. Once charting was completed, the authors compared their findings with their peers to determine agreements and conflicts, the latter of which was reconciled through discussion among all three investigators (KH, MG, and PB). Covidence calculated a Cohen’s kappa (K) coefficient statistic to measure the interrater reliability of the abstracts screened and full texts reviewed [6]. The primary author (PB) reviewed and adjudicated at each step of the review process and met with the research team in person for one hour each week (over 15 weeks) to provide direction and answer queries.

**Figure 1:**
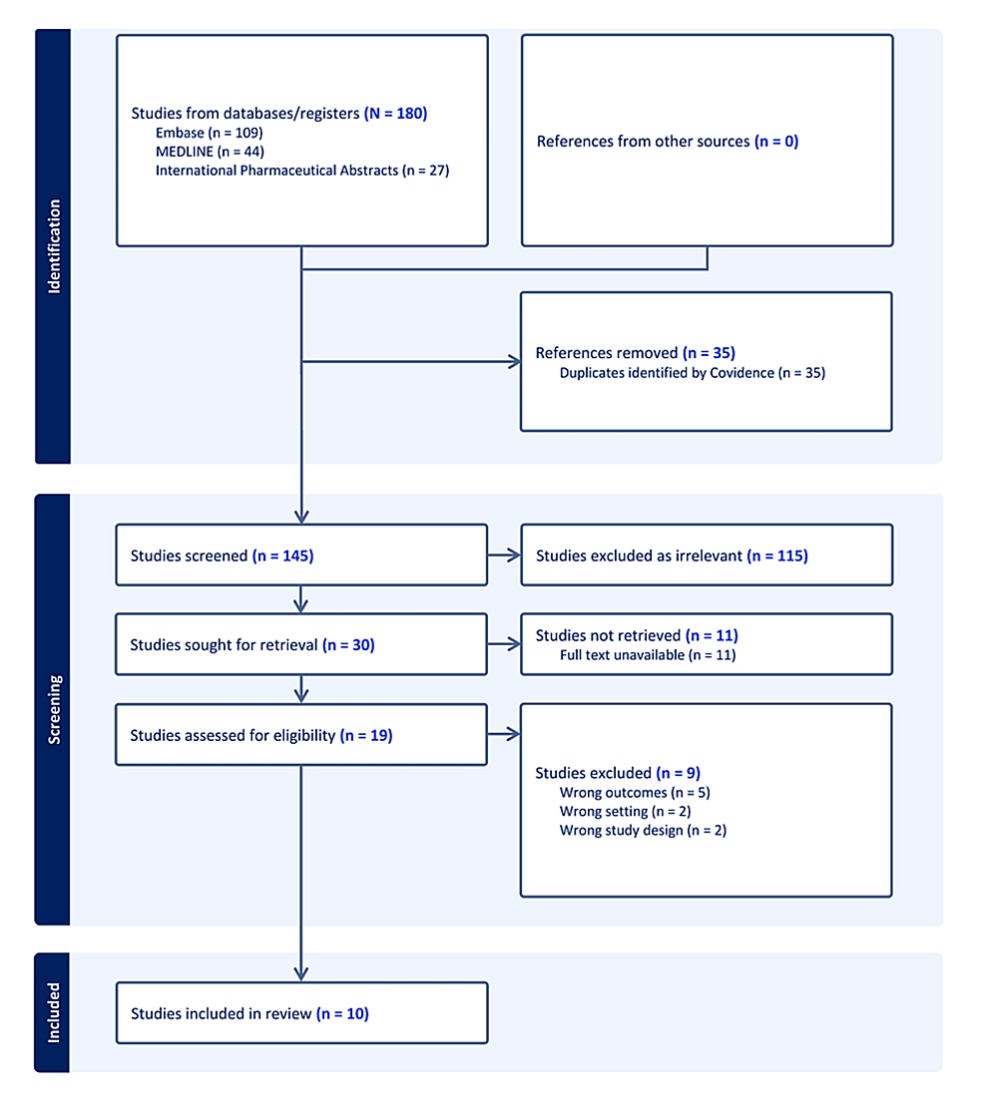
Flow diagram of record collection, abstract screening, full-text appraisal, and study inclusion COPD = chronic obstructive pulmonary disease

Figure 1 depicts the flow diagram for the search, screen, assessment, and inclusion of studies. One hundred forty-five unique articles were identified across the database searches and their abstracts were screened for eligibility. Thirty abstracts were deemed eligible and sought for full-text appraisal. Cohen’s kappa coefficient statistic for interrater reliability was 0.46 and 0.62 for the abstract and full-text screening, respectively [6].

Ten studies were ultimately included and are charted in Table 1 [7-16]. All studies were published in or after the year 2003 [7-16]. Over 80,000 patients living with COPD were represented across all 10 studies [7-16]; however, over 78,000 patients in this review were those identified in a population-based retrospective cohort of health administrative data in Ontario, Canada [13]. Four studies reported the average age of their sample was 70 years and older [7,11,14,16]. Five studies were conducted in the United States [8,10-12,16], two in Canada [9,13], and one study each in Australia [14], Palestine [7], and Spain [15]. The most common study design was that of a cohort trial (n=7) [7,10,11,13-16] and the most common institutional settings were community teaching hospitals (n=5) [7,10,11,14,16].

**Table 1:** Studies assessing medication errors during and following hospitalization for chronic obstructive pulmonary disease Abbreviations: aRR=adjusted risk ratio; AR=Arkansas; CCI=Charlson Comorbidity Index; CI=confidence interval; COPD=chronic obstructive pulmonary disease; CV=cardiovascular; DM=diabetes; DRP=drug-related problem; ECOPD=exacerbation of COPD; ED=emergency department; F=female; FEV1= forced expiratory volume in one second; h=hour(s); ICS=inhaled corticosteroid; IQR=interquartile range; LACE=length of stay, acuity of admission, comorbidity, emergency visit; LABA=long acting β agonist; LAMA=long-acting muscarinic antagonist; LOS=length of stay; M=male; MDI=meter dose inhaler; MN=Minnesota; MRP=medication-related problem; NC=North Carolina; NJ=New Jersey; OCS=oral corticosteroid; PIM=potentially inappropriate medication; PPO=potential prescribing omission; SABA=short-acting β agonist; SD=standard deviation

Author (year)	Location	Study Design and Setting	Population Characteristics	Medication Error Outcomes
Abukhalil (2022)[7]	West Bank, Palestine	Multicenter retrospective observational cohort of 2 hospitals	N=247; n=123 M; median age 73 years (IQR 67.8-80.4); median CCI 4 (IQR 3.1-6.2)	PPO: 4.8% of all PPOs related to the respiratory system PIM: 7 patients living with COPD prescribed OCS instead of ICS
Brown (2016)[8]	MN, United States	Pre-post study at a Veterans Affairs health care center	N=275; n=194 pre-implementation period (98.1% M; 49.4% predicted FEV1); n=81 post-implementation period (94.1% M; 52.8% predicted FEV1)	Errors pre-implementation of ECOPD order set upon discharge: SABA: 13.9%, LAMA or LAMA: 16.5%, ICS: 18%; Errors post-implementation of ECOPD order set upon discharge: SABA: 2.5% (p=.005 vs pre-implementation), LAMA or LABA: 7.4% (p=.047 vs pre-implementation), ICS: 9.9% (p=.089 vs pre-implementation)
Choi (2004)[9]	Toronto, Canada	Retrospective chart review at an academic medical center	N=105; mean age 76 years; n=56 F	Inpatient gaps in care: 89 inpatients ordered an MDI but 69 patients (78%) lacked documentation assessing appropriate inhaler technique; Discharge gaps in care: 98 patients were prescribed an inhaler on discharge and 71 patients (72%) did not receive inhaler education; 65 patients had their bronchodilator device changed before discharge but only 23 patients (35.4%) were appropriately observed for at least 24 h after device change; 103 patients were deemed eligible for ICS or OCS on discharge; and 18 patients (17.5%) did not receive a necessary ICS or OCS on discharge
Cooper (2020)[10]	NC, United States	Retrospective cohort at a community hospital	N=203; 43.8% F; median age 62.1 years (IQR 24-91); n=73 living with COPD	Cohort: 203 patients were discharged from the hospital during the study period and 19 patients (9.4%) had a COPD diagnosis as their reason for admission; MRP: 102 patients had an MRP and 9 patients (8.8%) had a COPD diagnosis; 6 MRPs in COPD patients (66.7%) were determined to be potentially preventable; All 6 potentially preventable MRPs in COPD patients (100%) pertained to ICS
Eisenhower (2014)[11]	United States	Prospective cohort at a community teaching hospital	N=25 inpatients admitted for ECOPD; n=21 not subsequently readmitted (mean age 74.7 years + 7, 38% M); n=4 patients readmitted (mean age 71.3 years + 6.1, 75% M)	Of 25 patients analyzed, there were 6 discrete medication discrepancies among 3 patients; Inpatient: 2 prescribing errors: inappropriate increase in fluticasone dose in fluticasone/salmeterol inhaler; Discharge: 1 transcribing error: omitted fluticasone/salmeterol on discharge reconciliation form
Evans (2020)[12]	AR, United States	Pilot study at an academic medical center	N=50 (mean age 63 years + 14, n=33 F); n=18 with COPD primary diagnosis	298 MRPs identified among 48 patients (96%) and included: omissions (n=97), unnecessary inappropriate therapy (n=94), wrong dose, frequency, or direction (n=69), duplicate therapy (n=38); Errors and interventions within COPD subgroup unreported
Gershon (2021)[13]	Ontario, Canada	Population-based retrospective cohort of health administrative data	N=78,953 highly-compliant medication users; n=69,253 LAMA users (n=18,330 hospitalizations and n=36,156 ED visits); n=36,439 LABA-ICS users (n=9,283 hospitalizations and n=19,901 ED visits); users compared against non-hospitalized controls (n=14,767 on LAMA and n=7,255 on LABA-ICS)	Hospitalized group: 5.2% of LAMAs were unintentionally discontinued (aRR 1.50 [95% CI 1.34-1.67; p < .001]), 5.5% of LABA-ICS were unintentionally discontinued (aRR 1.62 [95% CI 1.39-1.90; p < .001]), Hospitalization was associated with 50-62% increased risk of inhaled medications being inappropriately discontinued; Control (non-hospitalized) group: 3.3% of LAMAs were unintentionally discontinued; 3.1% of LABA-ICS were unintentionally discontinued
Peterson (2003)[14]	Tasmania, Australia	Multicenter retrospective cohort at community teaching hospitals	N=302; n=205 patients living with COPD (mean age 72 years; 61% M, 7-day LOS)	Hospitalized: 93.2% of COPD patients received nebulized ipratropium while hospitalized, and therapy was deemed inappropriate in 48% because both nebulized ipratropium plus inhaled ipratropium were administered; Discharge: 45% of COPD patients received nebulized ipratropium on hospital discharge, when inhaled ipratropium should have been prescribed; 32% of COPD patients received longer-than-necessary durations of ipratropium on hospital discharge, particularly in patients with asthma-COPD overlap
Romero-Ventosa (2018)[15]	Spain	Prospective cohort within an integrated primary/specialty/hospital care facility	N=58; 82.7% M; mean CCI 7.2	627 home treatments reconciled and 86.2% of patients had at least 1 MRP; Causes of MRPs: Need: 46%, Effectiveness: 27%, Safety: 27%
Singh (2021)[16]	NJ, United States	Retrospective cohort at a community hospital	N=115 (n=50 intervention vs n=65 control); mean age 70.3 years; 47.7% M; Mean LACE Index 9.2	Patients with medication reconciliation errors: Control n=37 (65.9%); Intervention pre-intervention n=27 (54%); Intervention post-intervention n=20 (40%); Medication reconciliation errors: Control n=45; omission (n=10), dose or frequency error (n=31), duplication (n=4) Intervention pre-intervention n=30; omission (n=13), dose or frequency error (n=14), duplication (n=3); Intervention post-intervention n=23; omission (n=12), dose or frequency error (n=8), duplication (n=3); Dose or frequency errors: SABA: control (n=3), pre-intervention (n=1), post-intervention (n=1) LABA or LAMA: control (n=27), pre-intervention (n=13), post-intervention (n=7); Omission errors: SABA: control (n=4), pre-intervention (n=5), post-intervention (n=5), LABA or LAMA: control (n=5), pre-intervention (n=6), post-intervention (n=6)

Medication-related problems

In a pilot study conducted at an academic medical center, pharmacists performing admission and discharge medication reconciliation discovered a medication-related problem (MRP) in 96% of patients during admission and discharge transitions of care, with an average of six MRPs encountered per patient [12]. These MRPs included omissions, unnecessary or inappropriate medications, duplicate therapy, and orders written with the wrong dose, frequency, or directions [12]. Thirty-six percent of patients in this pilot study were admitted with COPD as the reason for hospitalization but specific MRPs attributable to COPD hospitalizations were not provided [12]. In a prospective study conducted in Spain at a large integrated healthcare system, 25% of all MRPs were associated with inhaled medications and were deemed as being related to need, effectiveness, and safety [15]. This report did not, however, enumerate the counts and frequencies of need, effectiveness, and safety measures specific to inhaled medications [15]. A North Carolina community hospital conducted a retrospective chart review on MRPs in which six MRPs (5.9%) with ICS were all determined to be potentially preventable [10]. In a prospective cohort study of 25 patients at a teaching hospital, six MRPs were identified across four readmitted patients [11]. Among them, one MRP (16.7% of all MRPs) was a transcribing error involving fluticasone/salmeterol, wherein the inhaler was omitted from one patient’s pharmacotherapy plan, and in two other patients, prescribing errors occurred (i.e. one in each patient (33.4% of all MRPs)) when the fluticasone dose was inappropriately increased to 500 mcg twice daily [11].

One Veterans Affairs health care center conducted a pre-post study and found an absolute decrease of greater than 10% in prescribing errors upon discharge in each of the categories of SABA, LABA/LAMA, and ICS upon implementing an order set for ECOPD [8]. Decreases in the incidence of inhaled medication errors post-implementation were statistically significant (p<.05) versus pre-implementation for SABAs (2.5% vs 13.9%) and LABAs/LAMAs (7.4% vs 16.5%) but not ICS (9.9% vs 18%) [8]. A New Jersey community hospital conducted a retrospective cohort study and identified medication errors at discharge caused by dose or frequency errors decreased after having a pharmacist conduct a medication reconciliation at the time of discharge (14 vs 8, pre-post pharmacist intervention) [16]. Omissions of SABAs, LABAs, and LAMAs on discharge were unchanged (11 vs 11, pre-post pharmacist intervention) [16].

Prescribing errors and omissions

One retrospective cohort study of older adults hospitalized in Palestine leveraged the Screening Tool of Older Persons’ Prescriptions and Screening Tool to Alert to Right Treatment (STOPP/START) to determine potentially inappropriate prescribing via inappropriate medications and omissions [7]. The authors identified 221 discrete instances of potentially inappropriate prescribing, of which seven were in patients living with COPD who were prescribed an oral corticosteroid instead of an ICS [7]. Out of 247 patients in the sample, approximately 5% of all potential prescribing omissions occurred in patients with respiratory diseases such as COPD [7].

In one multisite retrospective cohort study conducted in Australia, 93.2% of hospitalized COPD patients received nebulized ipratropium, with 48% of these patients deemed as having received inappropriate nebulized ipratropium because they were ordered (duplicative) inhaled ipratropium concomitantly [14]. At discharge, 45% of patients were inappropriately ordered nebulized ipratropium when inhaled ipratropium (i.e. hydrofluoroalkane (HFA) device) should have been prescribed [14]. More than 30% of patients ordered ipratropium on discharge were prescribed a supply for longer than clinically necessary [14]. In one retrospective chart review of inpatients in Canada, 17.5% of eligible patients were not prescribed oral or inhaled steroid therapy upon discharge [9]. Prescribing omissions related to ICS, specifically, were not provided in this report and it is plausible that fewer than 17.5% of the eligible patients (not prescribed oral and inhaled steroids) had an ICS omitted from their treatment plan [9]. This study also reported that as many as 78% of patients admitted with ECOPD were prescribed an inhaler but may not have been taught or assessed on appropriate inhaler techniques [9]. One project capturing information from the Ontario Drug Benefit database conducted a population-based retrospective cohort study on medication discontinuation in patients living with COPD and found hospitalization was associated with a 50-62% increased risk of inhaled medications, specifically LAMA monotherapy and ICS/LABA combination therapy, being unintentionally discontinued compared to patients who were not hospitalized [13].

Discussion

The rate of inhaled medication errors was heterogeneous across the 10 studies in this review [7-16]. Brown and colleagues reduced the rate of SABA errors to as low as 2.5% after implementing an order set at discharge following ECOPD [8]. Peterson and colleagues determined that 45% of patients erroneously received nebulized ipratropium on hospital discharge [14] and between 40% and 66% of patients experienced errors via medication reconciliation in the study by Singh and colleagues [16]. Based on the studies included in this review, the rate of inhaled medication errors was heterogeneous and could lie anywhere between approximately 2% and 70% [7-16]. Future research, including population health analytics and case-control analyses, is needed to determine more homogenous estimates of medication errors. Studies conducted using administrative datasets, such as the project by Gershon and colleagues [13], may posit finite, accurate, and reasonable estimates of inhaled medication error rates, and similar international investigations should be undertaken. Five studies in this review highlighted errors occurring with an ICS [7-9,11,13]. Not all patients living with COPD are eligible for an ICS [1]. Patients who are most likely to benefit include those with comorbid asthma (or a history of asthma), frequent ECOPD, or eosinophilia [1]. Patients living with COPD who are inappropriately prescribed an ICS are at increased risk for adverse drug reactions such as oropharyngeal candidiasis and carry increased risks for pneumonia [1]. The 2023 GOLD Report provides ample guidance via narrative explanations, key summaries, and flowcharts to help prescribers choose the correct inhaled medications to treat COPD [1]. A plausible reason for inappropriate ICS prescribing could be prescriber familiarity with and the commercial availability of ICS/LABA combination therapies, which are recommended for all steps of asthma treatment [17,18]. The ICS/LABA combination inhalers were commercially available for at least five years before LAMAs and LABA/LAMAs entered the market [1]. Compared to North America, short-acting inhalers and oral bronchodilators are prescribed more often internationally than long-acting inhalers because short-acting and oral bronchodilators are substantially cheaper than long-acting inhalers [1]. Mitigation strategies to prevent ICS errors in patients living with COPD, such as electronic order sets, interdisciplinary rounds, and pharmacist-led medication reconciliation, have both nominally and statistically reduced ICS errors as reported by four studies in this review [8,11,12,16]. Three studies in this review reported inhaled medication errors as they pertained to MRPs [10,12,15]. The Pharmacy Quality Alliance (PQA) MRP Framework is an evidence-based tool and is familiar to many clinicians [19]. Only the study by Cooper and colleagues used a validated framework (ie, PQA) to categorize MRPs [10]. To improve medication error and intervention reporting consistency and transparency, future studies should strive to use validated and recognized frameworks. Beyond the PQA MRP, an additional instrument includes the National Coordinating Council for Medication Error Reporting and Prevention (NCC MERP) index [20]. The NCC MERP index is an easy-to-use tool that categorizes errors and identifies the degree of harm to the patient. By adhering to PQA MRP or NCC MERP, future studies will be standardized, and their results may be more accurately compared or aggregated in future analyses. Treatment for COPD has drastically evolved over the last 60 years [21]. Inhaled bronchodilators and corticosteroids first gained attention as therapeutic options in the 1990s and these inhaled medications replaced existing systemic treatments including antibiotics, sympathomimetics, and methylxanthines [21]. The GOLD group and initiative was formally established in 2001, and their recommendations supporting or refuting inhaled corticosteroids have changed often over the last 20 years as our knowledge of COPD pathophysiology has improved [1,21]. This may explain, to some extent, the heterogeneity of inhaled medications used to treat COPD across the 10 studies included in our review [7-16]. To improve transparency, further research should reference the GOLD Report or another clinical practice guideline with current recommendations (at the time the study was conducted) to provide context on inhaled medication appropriateness during the study period.

This literature review is not without limitations. Although the authors utilized a systematic approach to searching, assessing, and presenting the included studies, this literature review did not meet the technical standards of either a systematic or scoping review. Future systematic reviews on the themes and topics raised within this literature review are necessary to answer patient-specific questions. The authors did not perform a bias assessment of each article and doing so would have qualified the methodologic rigor of the included studies; although six studies were retrospective designs [7,9,10,13,14,16] and none were randomized controlled trials, it may be reasonable to infer that the included studies were of low-to-moderate methodologic quality [7-16]. Spirometry is required to accurately diagnose COPD [1]; none of the 10 studies in this review confirmed COPD using spirometry and thus there may be bias toward diagnostic momentum or misclassification [22,23]. All 10 articles included were published in or after 2003 [7-16], despite the search parameters permitting articles published as early as 1946. This is possible because (the term) COPD was coined in the early 2000s and it replaced previous terms of this respiratory disorder: chronic bronchitis and/or emphysema [21]. Although the latter two terms are currently included as sub-categories under the COPD medical subject heading, it is possible that the databases searched may index and misattribute studies between 1946 and 2000 using retired terms. A subsequent search of the literature using all possible keywords connoting COPD is necessary. The Cohen’s kappa coefficients calculated during the abstract (K=0.46) and full-text screening (K=0.62) are indicative of weak and moderate interrater reliability, respectively [6,23,24]. However, the authors ultimately resolved disagreements in screening, inclusion, and data abstraction via discussion and consensus-building in order to ensure the correctness of the data presented.

## Conclusions

As few as 2.5% or as many as 66% of patients living with COPD who are hospitalized may experience an error in their inhaled medication pharmacotherapy plan. Pharmacologic classes with reported errors include short- and long-acting beta-agonists, muscarinic antagonists, and inhaled corticosteroids. Standardization in categorizing and reporting inhaled medication errors is necessary in subsequent studies; the Pharmacy Quality Alliance Frameworks or Medication Error Reporting and Prevention Index are validated and widely used instruments that may be considered in future studies. Future research should aim to clearly compare and contrast findings across clinical settings and investigations and determine the true incidence of inhaled medication errors.

## Appendices

Appendix A: database search strings

Ovid MEDLINE

Search Executed: February 1, 2023

Search Set Included for Review: #17

Database: Ovid MEDLINE(R) and Epub Ahead of Print, In-Process, In-Data-Review & Other Non-Indexed Citations and Daily <1946 to January 31, 2023>

Search Strategy:

--------------------------------------------------------------------------------

1     "33011204".ui. (1)

2     "24847095".ui. (1)

3     1 or 2 (2)

4     exp Pulmonary Disease, Chronic Obstructive/ (65505)

5     (chronic$ adj2 obstruct$ adj2 (air$ or lung$ or pulmon$)).mp. (81493)

6     copd$1.mp. (57081)

7     or/4-6 (103954)

8     exp Medication Errors/ (19837)

9     ((medicat$ or drug$ or pharmac$) adj3 (error$ or reconcil$)).mp. (19890)

10     8 or 9 (24373)

11     7 and 10 (167)

12     Patient Discharge/ (38329)

13     discharg$.mp. (336032)

14     12 or 13 (336032)

15     11 and 14 (44)

16     ..l/ 15 lg=en (44)

17     remove duplicates from 16 (44)

18     3 not 17 (1)

***************************

Embase

Search Executed: February 14, 2023

Search Set Included for Review: #15

Database: Embase Classic+Embase <1947 to 2023 February 13>

Search Strategy:

--------------------------------------------------------------------------------

1     chronic obstructive lung disease/ (167999)

2     (chronic$ adj2 obstruct$ adj2 (air$ or lung$ or pulmon$)).mp. (181496)

3     copd$1.mp. (108571)

4     or/1-3 (198207)

5     exp medication error/ (23164)

6     ((medicat$ or drug$ or pharmac$) adj3 (error$ or reconcil$)).mp. (29366)

7     5 or 6 (31829)

8     4 and 7 (397)

9     exp hospital discharge/ (170808)

10     discharg$.mp. (622436)

11     (hospital$ to home$ or h2h or h 2 h).mp. (8937)

12     or/9-11 (628973)

13     8 and 12 (110)

14     ..l/ 13 lg=en (110)

15     remove duplicates from 14 (109)

***************************

International Pharmaceutical Abstracts

Search Executed: February 14, 2023

Search Set Included for Review: #14

Database: International Pharmaceutical Abstracts <1970 to January 2023>

Search Strategy:

--------------------------------------------------------------------------------

1     (chronic$ adj2 obstruct$ adj2 (air$ or lung$ or pulmon$)).mp. (2276)

2     copd$1.mp. (1663)

3     1 or 2 (2706)

4     ((medicat$ or drug$ or pharmac$) adj3 (error$ or reconcil$)).mp. (8958)

5     3 and 4 (29)

6     discharg$.mp. (8162)

7     (hospital$ to home$ or h2h or h 2 h).mp. (306)

8     6 or 7 (8402)

9     3 and 8 (111)

10     (error$ or erron$ or mistak$ or pharmacovigil$ or discrepan$).mp. (16387)

11     9 and 10 (9)

12     5 or 11 (31)

13     ..l/ 12 lg=en (27)

14     remove duplicates from 13 (27)

***************************
